# Dorsolateral Prefrontal Cortex Activity Predicts Responsiveness to Cognitive–Behavioral Therapy in Schizophrenia

**DOI:** 10.1016/j.biopsych.2009.04.036

**Published:** 2009-09-15

**Authors:** Veena Kumari, Emmanuelle R. Peters, Dominic Fannon, Elena Antonova, Preethi Premkumar, Anantha P. Anilkumar, Steven C.R. Williams, Elizabeth Kuipers

**Affiliations:** a Department of Psychology, Institute of Psychiatry, King's College London, United Kingdom; b Division of Psychological Medicine, Institute of Psychiatry, King's College London, United Kingdom; c Centre for Neuroimaging Sciences, Institute of Psychiatry, King's College London, United Kingdom; d National Institute for Health Research (NIHR) Biomedical Research Centre for Mental Health, South London and Maudsley National Health Service Foundation Trust, London, United Kingdom

**Keywords:** CBT, cerebellum, connectivity, DLPFC, fMRI, psychosis, schizophrenia

## Abstract

**Background:**

Given the variable response to cognitive–behavioral therapy (CBT) when added to antipsychotic medication in psychosis and the evidence for a role of pretherapy level of frontal lobe–based cognitive function in responsiveness to CBT in other disorders, this study examined whether pretherapy brain activity associated with working memory neural network predicts clinical responsiveness to CBT in schizophrenia.

**Methods:**

Fifty-two outpatients stable on medication with at least one distressing symptom of schizophrenia and willing to receive CBT in addition to their usual treatment and 20 healthy participants underwent functional magnetic resonance imaging during a parametric n-back task. Subsequently, 26 patients received CBT for psychosis (CBT+treatment-as-usual [TAU], 19 completers) for 6–8 months, and 26 continued with TAU alone (17 completers). Symptoms in both patient groups were assessed (blindly) at entry and follow-up.

**Results:**

The CBT+TAU and TAU-alone groups did not differ clinically or in performance at baseline. The CBT+TAU group showed significant improvement in relation to the TAU-alone group, which showed no change, at follow-up. Stronger dorsolateral prefrontal cortex (DLPFC) activity (within the normal range) and DLPFC–cerebellum connectivity during the highest memory load condition (2-back > 0-back) were associated with post-CBT clinical improvement.

**Conclusions:**

DLPFC activity and its connectivity with the cerebellum predict responsiveness to CBT for psychosis in schizophrenia. These effects may be mediated by PFC–cerebellum contributions to executive processing.

A number of randomized controlled trials (RCTs) have demonstrated that persistent positive symptoms, particularly delusions, and secondary disturbances such as depression, are improved by cognitive–behavioral therapy for psychosis (CBT-P) in patients with schizophrenia (1–3). These effects, however, are seen with modest effect sizes and present, to a noticeable degree, in only about 50% of such patients. Given the variable response to CBT-P, it is important to elucidate the predictors of CBT efficacy for psychosis.

Cognitive functions may play a role in CBT responsiveness across most disorders but perhaps more so in disorders, such as schizophrenia, which are characterized by cognitive impairment (4). Although skilled therapy adaptations attempt to compensate for such impairment, it may still hinder effectiveness of CBT, perhaps by impeding patients' ability to remember information discussed in sessions; to acquire new, more flexible thinking styles or coping strategies; or to generalize specific issues discussed in therapy to other situations in life. For example, in depression better executive functioning and problem-solving ability predict a more favorable clinical outcome following CBT, but not following treatment with antidepressant or placebo or waiting for CBT (5,6). Intact executive functioning also predicts a favorable response to CBT for generalized anxiety disorder (7). Similarly, initial cognitive flexibility predicted a better outcome in an RCT of CBT-P (8). In a recent study (9), neurocognitive deficits predicted poorer function overall in older psychotic patients but did not moderate CBT effects. The therapy in this study, however, was targeted specifically at social functioning rather than at distress and symptom reduction. In general, clinical responsiveness to CBT across disorders may relate to executive processes (5–8) modulated by the frontal lobes, especially the dorsolateral prefrontal cortex (DLPFC) (10–12).

This study tested the hypothesis, for the first time to our knowledge, that pretherapy DLPFC activity, elicited with a parametric n-back working memory (WM) task (13,14) and detected with functional magnetic resonance imaging (fMRI), would predict clinical responsiveness to CBT-P in schizophrenia. Given known associations between impaired cognition and disrupted DLPFC connectivity in schizophrenia (15), we also examined DLPFC connectivity with other brain regions as a predictor of responsiveness to CBT-P.

## Methods and Materials

### Participants and Design

This investigation involved three groups. Group 1 consisted of 26 outpatients with schizophrenia (16) who received CBT-P for 6–8 months in addition to their treatment-as-usual (TAU; CBT+TAU), Group 2 of 26 outpatients with schizophrenia who received TAU during the course of this investigation (TAU alone), and Group 3 of 20 healthy participants (HC) who did not have a clinical diagnosis (17).

All participants were right-handed and had no history of neurological conditions or head injury. All patients 1) had been on stable doses of antipsychotics for ≥2 years and on their present antipsychotic for >3 months, 2) received a rating of ≥60 on the Positive and Negative Symptoms scale (PANSS) (18) and reported at least one positive “distressing” symptom, and 3) wished/agreed to receive 6–8 months of CBT-P in addition to their usual drug treatment. Patients for both (CBT+TAU, TAU-alone) groups 1) were from the same geographic area, 2) had been identified by their psychiatrists as suitable for CBT-P, and 3) wished to receive CBT in addition to their usual treatment. Those accepted for CBT-P by the Psychological Interventions Clinic for Outpatients with Psychosis (PICuP), South London and Maudsley (SLAM) National Health Service (NHS) Foundation Trust went into the CBT+TAU group; others, matched to those in the CBT+TAU group as much as possible, went into the TAU-alone group. With the resources available to the SLAM NHS Foundation Trust, only approximately 10% of eligible patients are offered CBT-P. The final CBT+TAU group had 19 patients (consent withdrawal, *n* = 4; medication noncompliance before follow-up, *n* = 1; nonusable data, *n* = 2), and the TAU-alone group had 17 patients (consent withdrawal, *n* = 5; acutely unwell/admitted to a secure hospital before follow-up, *n* = 2; nonusable data, *n* = 2). Table 1 presents participants' characteristics. All participants provided written informed consent after the study procedures had been explained to them.

All participants underwent fMRI during the n-back task and clinical assessment at entry. The CBT+TAU group then received 6–8 months of CBT-P following a published manual (19) in a specialist clinical service (PICuP, SLAM NHS Foundation Trust). Therapy sessions were conducted weekly or fortnightly, as preferred by the patient, for up to 1 hour. Patients received an average of 16 sessions. All CBT interventions were formulation-driven and focused on the therapy goals of the patient. The therapists were supervised by one of two investigators (E.K. or E.R.P.). TAU-alone patients were followed up over the same period as CBT+TAU patients. Symptoms were rated in all patients, using the PANSS (18), at entry and then 6–8 months later by an experienced and independent psychiatrist (*DF*) who was blind to whether patients received CBT.

### fMRI Paradigm and Procedure

**Figure 1:**
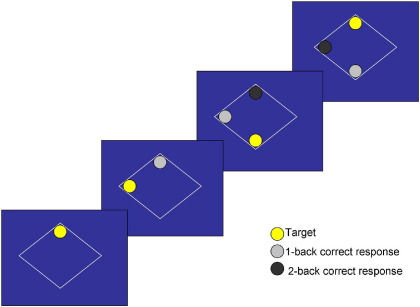
Illustration of 1-back and 2-back trials.

The task (14), modified from Callicott *et al.* (20), involved monitoring locations of dots (presentation time: 450 msec; interstimulus-interval: 1500 msec) within a diamond-shaped box on the screen at a given delay from the original occurrence (0-back, 1-back, or 2-back; Figure 1). There were three 30-sec active conditions (0-back, 1-back, 2-back) presented to participants five times in pseudo-random order, controlling for order effect. Each active block had 15 stimulus presentations, started with a 15-sec rest block (“Rest” on the screen), and began with a 750-msec text delay allowing the participants to notice a change in task demand/condition. The experiment lasted 11.25 min. Participants viewed the paradigm projected onto a screen through a prismatic mirror. They were required to press the button on every trial, using the right thumb, corresponding to the correct location of the 0-back, 1-back, or 2-back stimulus (chance performance = 25%; location of dots purely random). Participants abstained from alcohol for at least 24 hours and underwent task familiarization before scanning.

### Image Acquisition

Echoplanar MR brain images were acquired using a 1.5-T GE Signa system (General Electric, Milwaukee, Wisconsin). In each of 16 near-axial noncontiguous planes parallel to the intercommissural plane, 225 T2*-weighted MR images depicting blood oxygen level–dependent contrast were acquired over the experiment with echo time (TE) = 40 msec, repetition time (TR) = 3 sec, in-plane resolution = 3.1 mm, slice thickness = 7.0 mm, and interslice gap = .7 mm. In the same session, a high-resolution three-dimensional inversion recovery prepared spoiled gradient recalled acquisition in a steady state volume data set was acquired with TE = 5.3 msec, inversion time = 300 msec, TR = 12.2 msec, in-plane resolution = .94 mm, and slice thickness = 1.5 mm.

### Data Analysis: Demographic, Clinical, and Behavioral Measures

The HC, CBT+TAU, and TAU-alone groups were compared on age, education, and predicted IQ (21) using a one-way analysis of variance (ANOVA), followed by mean comparisons as appropriate. Group differences in performance were examined by a Group (HC, CBT+TAU, TAU-alone) × Load (0-back, 1-back, 2-back) ANOVA (separately for accuracy [% correct responses] and latency [in msec] of correct responses) with Group as a between-subjects factor and Load as the within-subjects factor, followed by analysis of lower order effects as appropriate. A significant Group effect in latency (Results), given the potential effect of age in this measure, was reevaluated using analyses of covariance (ANCOVA), covarying for age.

The CBT+TAU and TAU-alone groups were compared on clinical variables using independent-sample *t* tests. The change in symptoms from baseline to follow-up was investigated using a Group (CBT+TAU, TAU-alone) × Time (baseline, follow-up) ANOVA with Group as a between-subjects factor and Time as a within-subjects factor. A significant Group × Time effect was followed up by paired *t* tests on total and subscale PANSS scores separately in the CBT+TAU and TAU-alone groups. Following the observation of significant symptom reduction in the CBT+TAU group, but not in the TAU-alone group, we examined potential associations between baseline symptom severity and symptom change (baseline minus follow-up) in the CBT+TAU group using Pearson's correlations and confirmed the effects of CBT-P using ANCOVAs on symptom change scores covarying for baseline symptoms. We also computed the degree of change in symptoms independent of initial severity as residual change in symptoms by regressing the initial PANSS (total and subscales) scores on follow-up scores as a further outcome measure of CBT responsiveness for fMRI analysis following the method used by Siegle and colleagues (22). The association between performance variables and responsiveness to CBT was examined using Pearson's correlations.

All analyses were performed in SPSS windows (version 15). Before running the described analyses, each variable was evaluated for the normality of the distribution to ensure it met the criteria of parametric statistics. Alpha level for testing significance of effects was maintained at *p* < .05.

### Functional MRI: Image Pre-Processing

For each participant, the 225-volume functional time series were motion corrected, transformed into stereotactic space (Montreal Neurological Institute), smoothed with a 10 mm full-width-at-half-maximum Gaussian filter, and band-pass filtered using statistical parametric mapping software (SPM2; http://www.fil.ion.ucl.ac.uk/spm).

### Models and Statistical Inferences

Data were analyzed using a random-effect procedure (23). Subject-specific activations were identified with a factorial model consisting of three active conditions and rest as an implicit baseline. Generic task-related activity changes were identified using one-sample *t* tests (height threshold, *p* = .001; cluster-corrected *p* ≤ .05) separately in CBT+TAU and HC groups.

To examine the relationship of CBT response with pretherapy brain activity in patients, we regressed residual symptom change scores on task-related activations (0-back vs. rest; 1- and 2-back vs. 0-back) across the entire brain (height threshold *p* = .05, cluster-corrected *p* ≤ 05). For the positive associations of a priori hypothesized regions in the frontal lobe with CBT responsiveness, the following significance criteria to maxima voxels of clusters that did not survive whole-brain correction for multiple comparisons were applied: 1) *T* value of ≥3.80 (corresponding to uncorrected voxel *p* < .001) and ≥100 contiguous voxels, and 2) survival of small volume correction (SVC) within a locally defined volume (15-mm radius sphere) with family-wise error corrected *p* ≤ .05. (No cluster in any other regions met this SVC criterion for positive associations with CBT response.) We explored negative associations between CBT responsiveness and pretherapy activations using a more conservative criteria (height threshold, *p* = .005; whole-brain cluster-corrected *p* ≤ .05) because we did not have a specific hypothesis or a region of interest (ROI).

To examine whether a positive association between pretherapy brain activity and CBT responsiveness reflected a hyperresponse or a stronger response within the normal range in CBT+TAU patients, we compared CBT+TAU patients and HC using two-sample *t* tests on 2-back > 0 back contrasts (these revealed the strongest association with CBT responsiveness; see Results).

Further, we examined functional connectivity of the left and right frontal regions (that associated with CBT responsiveness in earlier analyses) with other regions as predictors of CBT responsiveness. For this purpose, the activity time series from the left DLPFC (seed −48[x], 34[y], 30[z]; this had the most consistent positive association with CBT response) and right inferior-middle PFC (seed 52[x], 24[y], and 22[z]) were extracted (2-back > 0-back) and used as a regressor (separately for left and right PFC) to investigate their connectivity with other regions. The regions with significantly covarying increases or decreases in activity with the two ROIs were identified for each participant, and the group connectivity maps constructed using one-sample *t* tests (height threshold *p* = .005; cluster-corrected *p* ≤ .05). The relationship of CBT response with connectivity of the left and right PFC with other regions was identified by regressing residual symptom change scores on connectivity SPM maps in CBT+TAU patients (height threshold *p* = .005; cluster-corrected *p* ≤ .05).

## Results

### Demographic, Clinical, and Behavioral Measures

There was a trend for the effect of Group in age (*F* = 2.44, *df* = 2,56, *p* = .10); TAU-alone patients were slightly older than HC (*t* = 1.96, *df* = 35, *p* = .06). There were Group effects in education (*F* = 3.53, *df* = 2,56, *p* = .04) and IQ (*F* = 4.70, *df* = 2,55, *p* = .01); TAU+ alone patients, relative to HC, had fewer years in education (*t* = 1.79, *df* = 35, *p* = .01) and lower IQ (*t* = 3.30, *df* = 34, *p* = .002). CBT+TAU patients did not differ from TAU-alone patients in age, education, IQ, baseline symptoms, age at illness onset, illness duration, and antipsychotic dose or from HC in demographic characteristics (Table 1).

CBT+TAU, but not TAU-alone, patients showed changes in symptoms from baseline to follow-up (Group × Time: total PANSS scores, *F* = 6.74, *df* = 1,34, *p* = .014; positive symptoms, *F* = 4.42, *p* = .043; negative symptoms, *F* = 4.47, *p* = .042; general psychopathology: *F* = 4.49, *p* = .041; Table 1). Only the CBT+TAU group showed reduced symptoms at follow-up (total PANSS scores: *t* = 3.43, *df* = 18, *p* = .003; positive symptoms: *t* = 3.48, *p* = .003; negative symptoms: *t* = 1.88, *p* = .07; general psychopathology: *t* = 3.02, *p* = .007). Baseline symptom severity did not correlate with CBT responsiveness (change in total symptoms, *r* = .17, *p* = .48). Covarying for baseline symptoms, there was significant symptom improvement (change scores) in the CBT+TAU, relative to TAU-alone, group (total PANSS scores: *F* = 8.16, *df* = 1,36, *p* = .007; positive symptoms: *F* = 7.01, *p* = .012; negative symptoms: *F* = 8.27, *p* = .007; general psychopathology: *F* = 5.98, *p* = .02). As can be expected given earlier noted independence between baseline symptoms and symptom improvement in CBT+TAU patients, residual symptom change score correlated highly positively with the absolute symptom change scores (total PANSS; *r* = .962, *p* < .001). Illness duration, education, and IQ were not associated with CBT responsiveness (*p* values > .40).

For performance accuracy, there was a main effect of Load (*F* = 91.85, *df* = 4,106, *p* < .001; lower accuracy with increasing load); Group (*F* = 1.86, *df* = 2,53, *p* = .17) and Group × Load (*F* = 1.66, *df* = 2,53, *p* = .20) effects were nonsignificant. For latency, there were significant Group (*F* = 4.24, *df* = 2,53, *p* = .02; covarying for age, *F* = 4.02, *df* = 2,52, *p* = .024) and Group × Load (*F* = 4.24, *df* = 2,53, *p* = .02; covarying for age, *F* = 4.02, *df* = 2,52, *p* = .024) effects indicating longer latencies in both patient groups compared to HC, especially at 1-back and 2-back (*p* values < .05); there was no difference between the CBT+TAU and TAU-alone groups (*p* values > .40). The relationships between CBT responsiveness and performance (accuracy: 1-back, *r* = .33, 2-back, *r* = .21; latency: *r* = −.08, *r* = −.25), although in the expected direction (better performance with positive CBT response), failed to reach significance.

### Generic fMRI Patterns

The generic WM network (Table 1 in Supplement 1) identified in both the CBT+TAU and HC groups included bilateral activations in the inferior-middle-superior frontal gyrus and the parietal lobe. The deactivated regions included the posterior cingulate, medial prefrontal and middle temporal gyri, insula, and precuneus.

### Pretherapy Brain Activity and CBT Responsiveness

#### Task-Related Activity Changes

**Figure 2:**
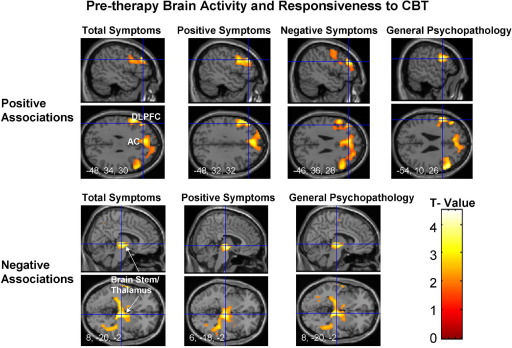
Brain activity associated with response to cognitive–behavioral therapy in patients with schizophrenia during the 2-back > 0-back contrast. The top row shows positive associations and the bottom row negative associations (maps thresholded at *p* = .05 uncorrected) in sagittal and transverse views with associated Montreal Neurological Institute coordinates (x, y, z). Left hemisphere is shown on the left of the transverse view. AC, anterior cingulate; CBT, cognitive-behavioral therapy; DLPFC, dorsolateral prefrontal cortex.

**Figure 3:**
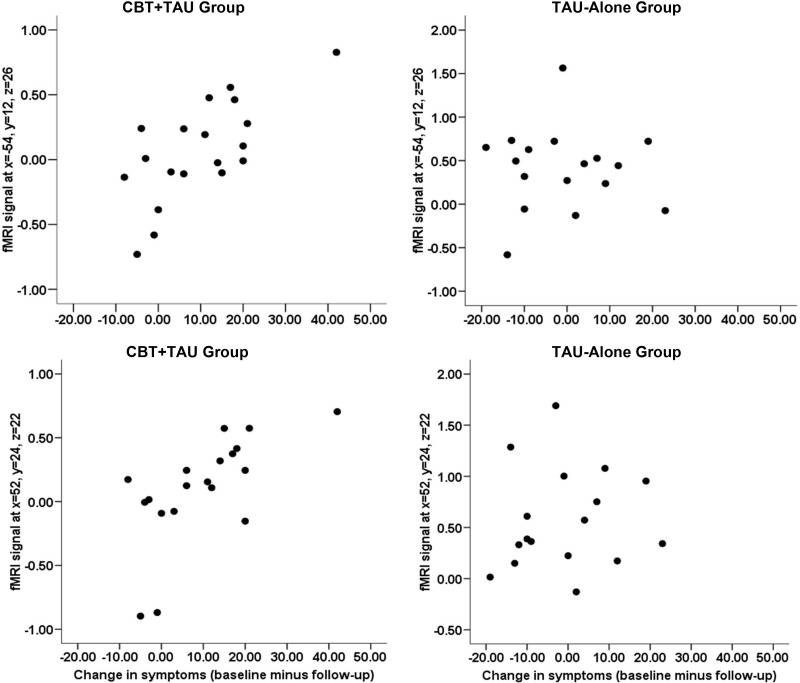
Scatterplots of task-load-related activity (2-back > 0-back) in the left (x= −54, y = 12, z = 26) and right (x = 52, y = 24, z = 22) frontal lobes against the change in symptoms (reduction from baseline) separately for the cognitive–behavioral therapy in addition to treatment-as-usual (CBT+TAU) and treatment-as-usual (TAU-alone) groups.

Expected associations emerged between pretherapy task-related activity at 2-back (> 0-back) and CBT responsiveness (Figure 2, Table 2); these relationships were absent in the TAU-alone group (Figure 3). Specifically, a reduction in total PANSS scores was associated with greater activity bilaterally in the inferior-middle frontal gyrus, mainly the DLPFC (BA 46). A medial PFC–anterior cingulate cluster (uncorrected *p* = .012) failed to survive SVC. A reduction in positive symptoms was associated with greater left inferior-middle frontal gyrus, most consistently Brodmann's area [BA] 9-46, activity. A reduction in negative symptoms was associated with greater pretherapy activity in a large left-sided cluster including the caudate, dorsomedial PFC, and DLPFC (BA 9-46). A reduction in general psychopathology was associated with greater activity bilaterally in the inferior-middle frontal gyrus, primarily BA 46.

No other activation was positively associated with a change in total or subscale symptom scores at any task load. Activity in several regions, mainly those found to be deactivated during memory load relative to no memory load (0-back/rest) conditions (Supplement 1), was associated negatively with CBT responsiveness during 1-back and 2-back (> 0-back) conditions (Figure 2, Table 2). These relationships are due to relatively stronger deactivations during memory load conditions in patients with the strongest CBT response.

The CBT+TAU and HC groups did not differ in activity of the regions positively or negatively associated with CBT responsiveness (*p* values > .20).

**Figure 4:**
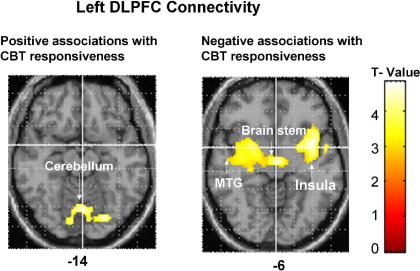
Left dorsolateral prefrontal cortex (DLPFC) connectivity association with response to cognitive–behavioral therapy (CBT) in patients with schizophrenia during the 2-back > 0-back contrast. The top row shows positive associations, and the bottom row shows negative associations (maps thresholded at *p* = .005 uncorrected) in transverse views with associated Montreal Neurological Institute z coordinates. Left hemisphere is shown on the left.

Within the left DLPFC connectivity maps (2-back > 0-back), CBT responsiveness associated positively with covarying increases in activity in a lingual gyrus-cerebellum cluster (peak: −4[*x*], −70[*y*], −4[*z*], *T* = 5.20; subpeaks: 8,−70, 0, *T* = 4.84 and −2,−70,−14; *T* = 4.30; 874 contiguous voxels; cluster-corrected *p* = .04), and negatively with activity in the insula extending to thalamus/brainstem and middle/superior temporal gyrus (peak: −38,6,−6; *T* = 5.04; subpeaks: −4,−14,−4; *T* = 4.74 and −40,−12,0, *T* = 4.67; 4187 contiguous voxels, cluster-corrected *p* < .001) (Figure 4). Within the right DLPFC connectivity maps (2-back > 0-back), CBT responsiveness associated negatively with activity in the thalamus extending to parahippocampal and posterior cingulate gyri (peak: −12,−24,6; *T* = 4.96; subpeaks: 14,−46,4; *T* = 4.83 and −4,−64,12; *T* = 4.65; 7841 contiguous voxels, cluster-corrected *p* < .001).

#### DLPFC Connectivity

In HC, the areas of significantly covarying increases with the left and right PFC activity during 2-back (> 0-back) included a large area surrounding the seed voxel and extending to the contralateral PFC and anterior cingulate, and a much smaller area in the left inferior parietal cortex (BA 40) (Table 2 in Supplement 1). Similar coactivations occurred in CBT+TAU patients, although the parietal cluster extended to a much smaller area for left DLPFC and was absent for right PFC. Posterior cingulate activity showed significantly covarying decreases with the left and right PFC activity in HC; this was present weakly for left PFC and nonsignificant for right PFC in CBT+TAU group.

## Discussion

We investigated the association between pre-CBT brain activity and responsiveness to 6–8 months of CBT-P in schizophrenia patients using an n-back task. We also studied a representative group of HC to enable us to characterize our patient sample.

### Clinical Findings

Supporting the findings of meta-analyses of RCTs for CBT-P (1–3), the CBT+TAU, relative to the TAU-alone, group showed reduced symptoms after CBT, with considerable variation in the degree of symptom change for individual patients (Figure 3).

### Neural Findings

The activation patterns in both the CBT+TAU and healthy groups are highly consistent with previous studies using this n-back task (13,14). Supporting our hypothesis, fMRI response increases in the frontal lobe, most consistently in the DLPFC, during the 2-back load predicted greater responsiveness to CBT. We found no associations between CBT responsiveness and 1) activations during 0-back condition and 2) task-related activations of the parietal cortex. The pattern of results suggests that cognitive processes attributed specifically to the frontal lobe, especially DLPFC, are more pertinent to CBT responsiveness in schizophrenia than WM in general or some other processes also engaged by the task (see below). Supporting this suggestion, the DLPFC activity (and not WM performance) explained a significant amount of variance in CBT responsiveness (*R*^2^ = .519, Standard error of the estimate = 9.33); the variance explained by fMRI activity and performance together (.520, 9.32) was similar to that explained by fMRI alone.

The task we used involves sustained attention, encoding of information into WM, active maintenance of stimulus representations, updating of sequential order information, and response inhibition and selection (20). Associations between task-related fMRI activity and CBT responsiveness could therefore be interpreted in terms of any of these functions. However, various brain regions constituting WM network are considered to subserve more specialized functions. The DLPFC (BA 9-46) contributes primarily to executive processes such as mnemonic strategies and monitoring (24,25) and executive control of maintenance and manipulation (26), rather than short-term storage of information (27). The DLPFC is also critical for relational processing in decision making (12), a function common to many higher-order processes such as reasoning and schema induction (28). Lateral PFC is implicated in top-down control to change behavior (29), and brain areas involved in the top-down processing of information are postulated to be associated with CBT responsiveness (30). The positive association we report here between DLPFC activity and CBT responsiveness may be mediated by facilitation of effective CBT by executive processes modulated by the DLPFC. Patients with greater DLPFC response may be more capable of schema induction (facilitating transfer of learning from one situation to other, similar, situations), reasoning, and relational processing (pooling together and comparing decision-relevant information) and gain most from CBT.

Previous studies have reported hyper-, hypo-, or normal-range frontal activations in schizophrenia depending on task characteristics, as well as clinical characteristics and performance of the patient groups (31–34). The use of atypical antipsychotics for most patients (35), and less marked (nonsignificant) deficit in performance accuracy in CBT+TAU patients, on average, compared with HC, possibly led to no patient-versus-control differences in WM-related activity in our study. The activations associated with CBT responsiveness in our sample did not reflect hyperactivations and were within the normal (healthy group) range. Recent data show that schizophrenia patients with normal-range (but not poor) performance, like healthy people, can show continued increases in DLPFC response from no/low-to-high memory load (34), as most likely is the case for patients with a good CBT-response in our study.

Although DLPFC activity of both hemispheres was predictive of CBT responsiveness, the left DLPFC showed more robust pattern of activity and connectivity with the cerebellum in association with CBT responsiveness. This suggests that the left hemisphere is more strongly associated with a beneficial outcome of CBT in schizophrenia, as reported previously in depression (36). In general, bilateral activity was expected with our task because it could be performed by encoding verbal (i.e., dot in the left/right/top/bottom position) or spatial information and was cognitively demanding (37).

The positive connectivity between the left DLPFC and cerebellum was strongly associated with a favorable response to CBT. Although the cerebellum has traditionally been implicated in motor control, there are stronger cerebellar projections from the PFC in humans (30.85%) than found in nonhuman primates (16.4%) (38), and these may have evolved in the course of evolution following the same course as the PFC itself (39). Furthermore, recent data demonstrate cerebellar contributions to higher-order cognitive functions, especially the task management and multitasking components of executive processing (40). The DLPFC-cerebellum connectivity and CBT responsiveness association may thus be explained by the PFC–cerebellum contributions to executive control, facilitating CBT responsiveness in the same way as the DLPFC activity itself. According to Andreasen *et al.* (41–43), disruption in the corticocerebellar-thalamo-cortical circuitry results in deficient processing, prioritizing, retrieval, coordination, and responding to information in schizophrenia. Our findings suggest that this circuitry may also have a role in responsiveness to CBT in schizophrenia.

Finally, we observed strong associations between a low, or no, response to CBT in patients and reduced deactivation of the regions that were deactivated during the rest/0-back, relative to the memory load, conditions in HC. These have generally been implicated in “mind-wandering” default states (44,45). Our finding may indicate an association between a reduced ability to maintain focus on, or switch to, a goal (task in this case) and a less favorable response to CBT. Clinically, disruption of default network activity has been reported in several disorders including autism (46), attention-deficit/hyperactivity disorder (47), and schizophrenia (48–51). Our findings suggest that default mode of brain action has a role in CBT efficacy in schizophrenia.

### Limitations

First, this study used a parallel-group, rather than a purely random, design for allocation to CBTp+TAU and TAU-alone groups. Although we cannot prove that the patients in the TAU group would also improve if they received CBT, the patients were randomly distributed across both groups in their desire for this intervention. Second, it could be argued that CBT+TAU patients showed clinical improvement simply because of benefiting from therapist contact, independent of the specific effects of the CBT methods applied to them. It is, however, unlikely because the standard care provided to patients before, and during, the study consisted of management offered by a case management team with a dedicated care coordinator who saw the patient regularly, in addition to psychiatrists and other specialists, such as a benefits adviser and occupational therapist. Furthermore, CBT for psychosis has specific effects on symptoms, distinct from interventions such as social skills training (help acquire social skills) or cognitive remediation (improve cognitive functioning) (1,3), and has been found superior in reducing the symptoms to a nonspecific befriending intervention controlling for the amount of contact with treating professionals (52). Third, the CBT+TAU and TAU-alone groups differed slightly in IQ, education, and illness duration; none of these, however, had a noticeable influence in CBT responsiveness. Fourth, the use of a block design limited the interpretation of fMRI findings in terms of the component processes involved in task performance. Finally, we employed a spatial MW task; verbal WM may be more pertinent to skills needed to engage in CBT.

### Conclusions

Within the WM network, the DLPFC activity and its connectivity with the cerebellum are associated with CBT responsiveness in schizophrenia. These effects may be mediated by the PFC–cerebellum contributions to executive processes facilitating effective CBT within a psychotherapeutic context. Our results may imply that addressing cognitive deficits associated with DLPFC in schizophrenia would maximize benefit from CBT. This is in line with recent data showing better outcomes with a combination of cognitive training and psychiatric rehabilitation in schizophrenia (53,54).

## Figures and Tables

**Table 1:** Demographics, Task Performance, and Clinical Characteristics of Participants

	Healthy Participants (n = 20; 14 men)	Patients	
		CBT+TAU Group (n = 19, 14 men)	TAU-Alone Group (n = 17, 15 men)
Demographics	Mean (SD)	Mean (SD)	Mean (SD)
Age (Years)	33.20 (11.28)	35.20 (7.79)	40.24 (10.34)
Education (Years)	15.55 (2.78)	13.79 (3.34)	13.35 (1.54)
Predicted IQ^a^	♢115.05 (7.53)	110.44 (9.75)	106.17 (8.63)
Performance	Mean (SEM)	Mean (SEM)	Mean (SEM)
Accuracy (%; Chance Performance = 25%)			
0-back	89.13 (2.87)	86.18 (2.95)	88.08 (2.68)
1-back	76.29 (4.23)	65.56 (4.34)	75.97 (4.99)
2-back	58.54 (5.29)	45.18 (5.43)	44.52 (5.88)
Reaction Time (msec)			
0-back	136.78 (14.26)	Δ209.07 (22.02)	Δ207.56 (25.97)
1-back	209.01 (31.80)	Δ222.52 (30.11)	Δ274.58 (42.51)
2-back	296.79 (43.96)	Δ562.23 (94.70)	Δ426.08 (58.77)

Patients Only						
	CBT+TAU Group Mean (SD)			TAU-alone Group Mean (SD)		
Clinical Variables	Baseline	Follow-up	Change (Baseline Minus Follow-up)	Baseline	Follow-up	Change (Baseline Minus Follow-up)
Age at Illness Onset (Years)	24.26 (7.91)			24.82 (8.43)		
Duration of Illness (Years)	10.74 (8.06)			15.41 (12.21)		
bPositive Symptoms	17.89 (4.86)	▾14.79 (4.39)	▴3.11 (3.88)	18.53 (3.10)	18.23 (3.67)	0.29 (4.13)
bNegative Symptoms	16.89 (3.52)	15.21 (4.04)	▴1.68 (3.90)	18.82 (3.94)	20.06 (4.53)	−1.24 (4.38)
bGeneral Psychopathology	32.95 (5.27)	▾28.05 (7.48)	▴4.89 (7.06)	34.82 (4.72)	34.76 (7.04)	0.06 (6.57)
bTotal Symptoms	67.74 (10.87)	▾58.05 (14.93)	▴9.68 (12.29)	72.17 (9.47)	73.06 (12.91)	−0.88 (12.07)
Antipsychotic medication	17 patients on atypical and 2 on both atypical and typical antipsychotics.	As baseline		15 patients on atypical and 2 on both atypical and typical antipsychotics.	As baseline	
Antipsychotic Dose in Chlorpromazine Equivalents (mg)	544.00 (426.46)	As baseline		474.68 (338.70)	As baseline	

Duration of illness = current age minus age of illness onset; CBT, cognitive-behavioral therapy; TAU, treatment-as-usual.

a National Adult Reading Test (21);

b Positive and Negative Symptom Scale (18);

♢ N = 19 (missing IQ data in one healthy participant) and IQ in healthy participants significantly higher than TAU-alone;

Δ Longer latencies (*p* < .05) in patients relative to healthy participants even when controlling for age;

▾ Lower symptom scores (*p* < .05) at follow-up in the CBT+TAU, but not in the TAU-alone, group;

▴ Symptom reduction (*p* < .05) in the CBT+TAU, relative to the TAU-alone, group.

**Table 2:** Brain Areas Showing Positive and Negative Associations with CBT Responsiveness in Patients

Positive Associations	BA	Cluster Size (voxels)	Side	MNI Coordinates			Voxel T Value	Corrected *p*
				X	Y	Z		
Total Symptoms								
2-back > 0-back								
Inferior-middle frontal gyrus	44	1048	L	−54	12	26	4.85	*.015*
	46		L	−48	34	30	4.43	
Inferior-middle frontal gyrus	45/46	1132	R	52	24	22	3.99	*.033*
	46		R	42	24	26	2.95	
Positive Symptoms								
2-back > 0-back								
Inferior-middle frontal gyrus	9–46	1263	L	−48	32	32	4.61	*.021*
	44		L	−52	12	28	4.03	
	9		L	−54	22	32	3.75	
Negative Symptoms								
2-back > 0-back								
Caudate nucleus	n/a	12802	L	−14	2	14	5.60	<.001
Anterior cingulate/medial prefrontal cortex	32/8		L	−6	22	52	4.57	
Middle frontal gyrus	9–46		L	−46	36	28	4.55	
General Psychopathology								
2-back > 0-back								
Inferior-middle frontal gyrus	44	874	L	−54	10	26	4.51	*.025*
	46		L	−48	34	30	3.83	
Middle frontal gyrus	46	723	R	52	26	24	4.48	*.026*

Negative Associations								
Total Symptoms								
1-back > 0-back								
Insula	n/a	15746	L	−30	−28	6	5.50	<.001
Middle temporal gyrus	20/21		L	−36	−34	−14	5.32	
Parahippocampal gyrus	36		L	−20	−40	−14	4.65	
2-back > 0-back								
Brain stem	n/a	7729	L	−8	−20	−2	3.83	.006
Middle temporal gyrus	21		L	−60	0	−10	3.65	
Inferior parietal lobe	40		L	−50	−40	48	3.54	
Positive Symptoms								
1-back > 0-back								
Transverse temporal gyrus	41	11704	L	−30	−30	10	4.94	<.001
Posterior cingulate	23		R	10	−36	28	4.91	
	23/31		R	14	−56	20	4.82	
2-back > 0-back								
Brain stem	n/a	6593	R	6	−18	−2	4.24	.016
	n/a		R	8	−12	−8	3.92	
Middle temporal gyrus	39		R	36	−62	18	3.60	
Negative Symptoms								
None								
General Psychopathology								
1-back > 0 back								
Middle temporal gyrus	21/20	16245	L	−38	−32	−14	5.31	<.001
Parahippocampal gyrus	35/36		L	−18	−38	−14	5.14	
Fusiform gyrus	37		L	−32	−40	−16	5.02	
2-back > 0-back								
Brain stem	n/a	17435	R	8	−20	−2	4.37	<.001
Posterior cingulate	31		L	−24	−60	18	4.09	
Inferior parietal lobe	5		L	−24	−44	54	3.94	

BA, Brodmann area; MNI, Montreal Neurological Institute. In italics: SVC criterion applied. All others: cluster *p* corrected for multiple comparisons across the entire brain.

## References

[1] Pfammatter M., Junghan U.M., Brenner H.D. (2006). Efficacy of psychological therapy in schizophrenia: Conclusions from meta-analyses. Schizophr Bull.

[2] Wykes T., Steel C., Everitt B., Tarrier N. (2008). Cognitive behavior therapy for schizophrenia: Effect sizes, clinical models, and methodological rigor. Schizophr Bull.

[3] Pilling S., Bebbington P., Kuipers E., Garety P., Geddes J., Orbach G. (2002). Psychological treatments in schizophrenia: I. Meta-analysis of family intervention and cognitive behaviour therapy. Psychol Med.

[4] Reichenberg A., Harvey P.D. (2007). Neuropsychological impairments in schizophrenia: Integration of performance-based and brain imaging findings. Psychol Bull.

[5] Moorey S., Holting C., Hughes P., Knynenberg P., Michael A. (2001). Does problem solving ability predict therapy outcome in a clinical setting?. Behav Cognit Psychother.

[6] Julian L.J., Mohr D.C. (2006). Cognitive predictors of response to treatment for depression in multiple sclerosis. J Neuropsychiatr Clin Neurosci.

[7] Mohlman J., Gorman J.M. (2005). The role of executive functioning in CBT: A pilot study with anxious older adults. Behav Res Ther.

[8] Garety P., Fowler D., Kuipers E., Freeman D., Dunn G., Bebbington P. (1997). London-East Anglia randomised controlled trial of cognitive-behavioural therapy for psychosis: II: predictors of outcome. Br J Psychiatry.

[9] Granholm E., McQuaid J.R., Link P.C., Fish S., Patterson T., Jeste D.V. (2008). Neuropsychological predictors of functional outcome in cognitive behavioral social skills training for older people with schizophrenia. Schizophr Res.

[10] Cabeza R., Nyberg L. (2000). Imaging cognition II: An empirical review of 275 PET and fMRI studies. J Cogn Neurosci.

[11] Duncan J., Owen A.M. (2000). Common regions of the human frontal lobe recruited by diverse cognitive demands. Trends Neurosci.

[12] Krawczyk D.C. (2002). Contributions of the prefrontal cortex to the neural basis of human decision making. Neurosci Biobehav Rev.

[13] Kumari V., Aasen I., Taylor P., Ffytche D.H., Das M., Barkataki I. (2006). Neural dysfunction and violence in schizophrenia: An fMRI investigation. Schizophr Res.

[14] Kumari V., Aasen I., Ffytche D., Williams S.C., Sharma T. (2006). Neural correlates of adjunctive rivastigmine treatment to antipsychotics in schizophrenia: A randomized, placebo-controlled, double-blind fMRI study. Neuroimage.

[15] Yoon J.H., Minzenberg M.J., Ursu S., Walters R., Wendelken C., Ragland J.D. (2008). Association of dorsolateral prefrontal cortex dysfunction with disrupted coordinated brain activity in schizophrenia: Relationship with impaired cognition, behavioral disorganization, and global function. Am J Psychiatry.

[16] First M.B., Spitzer R.L., Gibbon M., Williams J.B.W. (1995). Structured Clinical Interview for DSM-IV Axis I Disorders, Patient Edition (SCID-P), Version 2.

[17] First M.B., Spitzer R.L., Gibbon M., Williams J.B.W. (2002). Structured Clinical Interview for DSM-IV-TR Axis I Disorders, Research Version, Non-Patient Edition.

[18] Kay S.R., Fiszbein A., Opler L.A. (1987). The positive and negative syndrome scale (PANSS) for schizophrenia. Schizophr Bull.

[19] Fowler D., Garety P.A., Kuipers E. (1995). Cognitive Behaviour Therapy for Psychosis: Theory and Practice.

[20] Callicott J.H., Mattay V.S., Bertolino A., Finn K., Coppola R., Frank J.A. (1999). Physiological characteristics of capacity constraints in working memory as revealed by functional MRI. Cereb Cortex.

[21] Nelson H., Willison J. (1991). National Adult Reading Test Manual.

[22] Siegle G.J., Carter C.S., Thase M.E. (2006). Use of FMRI to predict recovery from unipolar depression with cognitive behavior therapy. Am J Psychiatry.

[23] Friston K.J., Holmes A.P., Worsley K.J. (1999). How many subjects constitute a study?. Neuroimage.

[24] Wagner A.D., Maril A., Bjork R.A., Schacter D.L. (2001). Prefrontal contributions to executive control: fMRI evidence for functional distinctions within lateral prefrontal cortex. Neuroimage.

[25] Chase H.W., Clark L., Sahakian B.J., Bullmore E.T., Robbins T.W. (2008). Dissociable roles of prefrontal subregions in self-ordered working memory performance. Neuropsychologia.

[26] Maestu F., Campo P., Capilla A., Simos P.G., Paul N., Fernandez S. (2005). Prefrontal brain magnetic activity: Effects of memory task demands. Neuropsychology.

[27] Honey G.D., Bullmore E.T., Sharma T. (2000). Prolonged reaction time to a verbal working memory task predicts increased power of posterior parietal cortical activation. Neuroimage.

[28] Waltz J., Knowlton B., Holyoak K., Boone K., Mishkin F., de Menedezes (1999). A system for relational reasoning in human prefrontal cortex. Psychol Sci.

[29] Garavan H., Ross T.J., Murphy K., Roche R.A., Stein E.A. (2002). Dissociable executive functions in the dynamic control of behavior: Inhibition, error detection, and correction. Neuroimage.

[30] van der Gaag M. (2006). A neuropsychiatric model of biological and psychological processes in the remission of delusions and auditory hallucinations. Schizophr Bull.

[31] Manoach D.S. (2003). Prefrontal cortex dysfunction during working memory performance in schizophrenia: Reconciling discrepant findings. Schizophr Res.

[32] Macdonald A.W., Thermenos H.W., Barch D.M., Seidman L.J. (2008). Imaging genetic liability to schizophrenia: Systematic review of fMRI studies of patients' nonpsychotic relatives. Schizophr Bull.

[33] Jansma J.M., Ramsey N.F., van der Wee N.J., Kahn R.S. (2004). Working memory capacity in schizophrenia: A parametric fMRI study. Schizophr Res.

[34] Karlsgodt K.H., Sanz J., van Erp T.G., Bearden C.E., Nuechterlein K.H., Cannon T.D. (2009). Re-evaluating dorsolateral prefrontal cortex activation during working memory in schizophrenia. Schizophr Res.

[35] Davis C.E., Jeste D.V., Eyler L.T. (2005). Review of longitudinal functional neuroimaging studies of drug treatments in patients with schizophrenia. Schizophr Res.

[36] Bruder G.E., Stewart J.W., Mercier M.A., Agosti V., Leite P., Donovan S. (1997). Outcome of cognitive-behavioral therapy for depression: Relation to hemispheric dominance for verbal processing. J Abnorm Psychol.

[37] Wager T.D., Smith E.E. (2003). Neuroimaging studies of working memory: A meta-analysis. Cogn Affect Behav Neurosci.

[38] Ramnani N., Behrens T.E., Johansen-Berg H., Richter M.C., Pinsk M.A., Andersson J.L. (2006). The evolution of prefrontal inputs to the corticopontine system: Diffusion imaging evidence from macaque monkeys and humans. Cereb Cortex.

[39] Ramnani N. (2006). The primate corticocerebellar system: Anatomy and function. Nat Rev Neurosci.

[40] Bellebaum C., Daum I. (2007). Cerebellar involvement in executive control. Cerebellum.

[41] Andreasen N.C., O'Leary D.S., Cizadlo T., Arndt S., Rezai K., Ponto L.L. (1996). Schizophrenia and cognitive dysmetria: A positron-emission tomography study of dysfunctional prefrontal-thalamic-cerebellar circuitry. Proc Natl Acad Sci U S A.

[42] Andreasen N.C., Paradiso S., O'Leary D.S. (1998). “Cognitive dysmetria” as an integrative theory of schizophrenia: A dysfunction in cortical-subcortical-cerebellar circuitry?. Schizophr Bull.

[43] Andreasen N.C., Nopoulos P., O'Leary D.S., Miller D.D., Wassink T., Flaum M. (1999). Defining the phenotype of schizophrenia: Cognitive dysmetria and its neural mechanisms. Biol Psychiatry.

[44] Greicius M.D., Krasnow B., Reiss A.L., Menon V. (2003). Functional connectivity in the resting brain: A network analysis of the default mode hypothesis. Proc Natl Acad Sci U S A.

[45] Raichle M.E., MacLeod A.M., Snyder A.Z., Powers W.J., Gusnard D.A., Shulman G.L. (2001). A default mode of brain function. Proc Natl Acad Sci U S A.

[46] Kennedy D.P., Redcay E., Courchesne E. (2006). Failing to deactivate: Resting functional abnormalities in autism. Proc Natl Acad Sci U S A.

[47] Tian L., Jiang T., Wang Y., Zang Y., He Y., Liang M. (2006). Altered resting-state functional connectivity patterns of anterior cingulate cortex in adolescents with attention deficit hyperactivity disorder. Neurosci Lett.

[48] Williamson P. (2007). Are anticorrelated networks in the brain relevant to schizophrenia?. Schizophr Bull.

[49] Buckner R.L., Andrews-Hanna J.R., Schacter D.L. (2008). The brain's default network: Anatomy, function, and relevance to disease. Ann NY Acad Sci.

[50] Garrity A.G., Pearlson G.D., McKiernan K., Lloyd D., Kiehl K.A., Calhoun V.D. (2007). Aberrant “default mode” functional connectivity in schizophrenia. Am J Psychiatry.

[51] Pomarol-Clotet E., Salvador R., Sarro S., Gomar J., Vila F., Martinez A. (2008). Failure to deactivate in the prefrontal cortex in schizophrenia: Dysfunction of the default mode network?. Psychol Med.

[52] Sensky T., Turkington D., Kingdon D., Scott J.L., Scott J., Siddle R. (2000). A randomized controlled trial of cognitive-behavioral therapy for persistent symptoms in schizophrenia resistant to medication. Arch Gen Psychiatry.

[53] McGurk S.R., Twamley E.W., Sitzer D.I., McHugo G.J., Mueser K.T. (2007). A meta-analysis of cognitive remediation in schizophrenia. Am J Psychiatry.

[54] Patterson T.L., Leeuwenkamp O.R. (2008). Adjunctive psychosocial therapies for the treatment of schizophrenia. Schizophr Res.

