# Supplementary material for: Dorsolateral Prefrontal Cortex Activity Predicts Responsiveness to Cognitive–Behavioral Therapy in Schizophrenia
Source: Biol Psychiatry. 2009 Sep 15;66(6):594–602. doi: 10.1016/j.biopsych.2009.04.036 (PMC2734077; doi:10.1016/j.biopsych.2009.04.036)
Supplement: Supplement 1 [file mmc1.pdf]

Supplemental Table 1. Brain areas showing activation increases and decreases in association with individual task conditions (height threshold *p* <.001) in patients (CBT+TAU) and healthy participants.

**Patients**	BA	Cluster size (voxels)	Side	MNI Coordinates			Voxel T value	Cluster *p* (corrected)
				X	Y	Z		
**0-back > Rest**								
Middle frontal gyrus	6	3658	L	-34	-10	60	11.22	<.001
Post-central gyrus	1/2		L	-40	-36	54	10.57	
Pre-central gyrus	4		L	-42	-18	60	9.43	
Medial frontal gyrus	6	609	R	2	0	58	8.61	.001
AC	32		R	6	6	48	6.43	
Medial frontal gyrus	6		n/a	0	-8	60	5.80	
Post-central gyrus	2/4/43	281	L	-58	-22	18	7.62	.036
**1-back > Rest**								
Pre-central gyrus	4	6398	L	-42	-20	58	10.86	<.001
Medial frontal gyrus/AC	6/32		L	-2	6	56	10.12	
Inferior parietal lobe	40		L	-38	-38	56	9.81	
Precuneus	7	921	R	12	-68	52	6.85	<.001
Inferior parietal lobe	40		R	46	-42	60	5.90	
Superior parietal lobe	7		R	36	-62	52	4.32	
Middle temporal gyrus	21	268	R	60	-62	0	6.66	.044
Inferior temporal gyrus	37		R	52	-74	2	4.63	
Superior-middle frontal gyrus	6	361	R	32	-6	58	6.65	.015
	6		R	42	0	54	6.21	
**2-back > Rest**								
Inferior parietal cortex	40	9671	L	-44	-44	50	12.25	<.001
Medial frontal gyrus/AC	6		L	-4	8	56	12.10	
Superior parietal lobe	7		L	-12	-68	56	11.98	
Superior-middle frontal gyrus	6	1996	R	42	0	52	8.32	<.001
(extends to sensorimotor cortex)	6		R	30	-2	58	6.72	
Middle frontal gyrus (DLPFC)	46		R	42	38	28	6.58	
Cerebellum	n/a	334	L	-36	-52	-36	7.76	.019
			L	-28	-60	-32	5.75	
		316	R	32	-54	-30	7.43	.023
			R	4	-66	-18	3.92	
Middle temporal gyrus	21	577	R	58	-64	0	5.95	.001
	37		R	50	-68	2	5.72	
Inferior temporal gyrus	37		R	50	-58	-14	5.67	
Middle frontal gyrus (DLPFC)	46	289	L	-40	50	20	5.51	.032
	10/46		L	-34	60	10	4.63	
Middle frontal gyrus	10		L	-40	56	2	4.54	
**1-back > 0-back**								
Middle frontal gyrus (DLPFC)	46	686	L	-44	32	26	7.77	<.001
	46		L	-36	44	22	6.45	
	9		L	-52	26	34	5.54	
Superior-middle frontal gyrus	6	599	L	-26	4	56	7.27	.001
	6/9		L	-46	4	46	6.80	
	6		L	-36	-4	52	6.03	

Superior-middle frontal gyrus (includes DLPFC)	6	1079	R	38	0	54	7.14	<.001
	8		R	30	6	52	5.49	
	10/46		R	42	48	22	5.33	
Occipital gyrus	19	2102	L	-26	-86	30	6.39	<.001
Precuneus	7		L	-8	-66	54	6.17	
Superior parietal lobule	7		R	16	-76	58	6.08	
**2-back > 0-back**								
Precuneus	7	4540	R	8	-62	56	9.69	<.001
Inferior parietal lobe	40		R	50	-40	48	9.19	
Precuneus	7		L	-10	-66	56	9.00	
AC	32/6	3442	R	4	20	52	8.45	<.001
Middle frontal gyrus (includes DLPFC)	9		R	52	14	32	8.30	
	46/10		R	38	52	20	7.32	
Middle frontal gyrus (includes DLPFC)	9/46	1987	L	-44	32	30	8.21	<.001
	10		L	-34	60	6	7.23	
	6		L	-24	4	56	6.76	
Inferior parietal lobe	40	827	L	-46	-46	46	7.24	<.001
	40		L	-36	-48	40	6.13	
	40		L	-26	-52	44	5.10	
**0-back > 1-back**								
Posterior cingulate	29/30	414	L	-2	-50	22	5.45	.004
	31		L	-10	-42	36	4.29	
	31		R	6	-50	32	3.94	
Paracentral lobule	7	242	L	-2	-36	54	4.79	.04
	5		R	8	-28	52	4.40	
	5		L	-2	-24	50	4.17	
**0-back > 2-back**								
Superior temporal gyrus	22	1271	R	60	-6	4	10.26	<.001
Insula	n/a		R	44	-4	14	5.42	
Middle temporal gyrus	21		R	56	-8	-16	4.78	
Medial prefrontal gyrus	10	1702	L	-4	58	-4	8.97	<.001
	9/10		L	-2	50	20	6.81	
	10/32		L	-4	48	10	6.66	
Posterior cingulate	31	3344	n/a	0	-22	46	8.49	<.001
	30/23		L	-6	-52	10	8.15	
	30/23		L	-10	-56	4	8.10	
Transverse temporal gyrus	41	613	L	-38	-24	10	5.91	.002
	41		L	-54	-20	14	4.82	
Superior temporal gyrus	22		L	-58	-6	10	4.33	
**Rest > 0-back**								
Thalamus	n/a	17375	R	26	-30	4	10.41	<.001
Parahippocampal gyrus	36/37		R	30	-30	-12	9.46	
Superior-middle temporal gyrus	21/22		R	36	-30	4	9.04	
**Rest > 1-back**								
Parahippocampal gyrus (extends to posterior cingulate)	36	18996	L	-36	-30	-10	8.44	<.001
Transverse temporal gyrus	41		L	-22	-32	22	8.20	
	41		R	28	-32	12	8.12	
Medial prefrontal gyrus	9	653	L	-18	52	22	6.16	.001
	9		L	-4	44	22	4.67	

	9/10		R	10	54	20	4.35	
**Rest > 2-back**								
Posterior cingulate	29	21550	L	-12	52	2	13.06	<.001
Post-central gyrus	40		R	42	-16	24	11.32	
Parahippocampal gyrus (extends to posterior cingulate)	23/31		L	-10	-54	26	10.31	
Medial prefrontal gyrus	10	1938	n/a	0	50	14	6.17	<.001
	9		R	10	50	24	6.06	
AC (Ventral)	24		n/a	0	28	18	6.03	
Middle temporal gyrus	39	390	L	-44	-64	28	5.86	.010
Inferior parietal lobe	40		L	-46	-74	40	5.33	
**Healthy Participants**								
**0-back > Rest**								
Pre-central gyrus	4	10168	L	-32	-24	56	14.27	<.001
	4		L	-48	-18	50	13.72	
Medial frontal gyrus	6		L	-12	-8	58	12.51	
Inferior parietal lobe	40	2215	R	34	-52	58	7.70	<.001
	40		R	38	-44	52	7.24	
	40		R	36	-42	44	6.62	
Putamen	n//a	559	L	-20	12	2	6.90	.004
Caudate/Putamen	n/a		L	-14	6	6	5.66	
Putamen	n/a		L	-20	2	10	4.95	
Middle occipital gyrus	19	553	L	-42	-66	2	6.75	.004
Middle occipital gyrus	19	654	R	44	-68	2	5.72	.002
Inferior occipital/temporal gyrus	18/19		R	54	-70	-6	5.53	
Middle occipital gyrus	19		R	44	-70	-8	5.40	
Cerebellum	n/a	1111	R	6	-54	-18	5.62	<.001
	n/a		R	16	-52	-26	5.50	
	n/a		R	26	-52	-32	5.44	
**1-back > Rest**								
Middle frontal gyrus	6	15985	L	-4	2	54	17.19	<.001
Pre-central gyrus	4		L	-34	-22	56	14.77	
	4		L	-44	-16	56	14.70	
Inferior parietal lobe	40	9632	R	40	-44	50	9.56	<.001
	40		R	38	-36	40	9.37	
Precuneus	7		R	14	-62	54	9.19	
Middle frontal gyrus (DLPFC)	46	834	R	38	38	30	8.56	.001
Putamen	n/a	344	R	18	2	8	7.32	.034
Middle frontal gyrus (DLPFC)	46	377	L	-42	36	30	6.40	.025
**2-back > Rest**								
Medial frontal gyrus/AC (to superior-middle frontal gyrus)	6	26041	L	-6	4	54	14.42	<.001
Inferior parietal lobe	40		R	40	-42	42	14.25	
	40		L	-46	-40	46	14.11	
Cerebellum	n/a	1888	R	4	-56	-20	9.18	<.001
			L	-24	-62	-34	6.88	

			R	26	-52	-34	5.76	
Inferior frontal gyrus/Insula	45	1005	L	-32	22	0	7.79	<.001
Thalamus	n/a		L	-14	-8	12	6.69	
Middle frontal gyrus (DLPFC)	9	526	L	-38	32	34	6.87	.007
Putamen	n/a	333	R	18	0	10	6.57	.038
Inferior frontal gyrus/Insula	47	448	R	36	20	-2	6.25	.013
	47		R	24	26	2	6.04	
**1-back > 0-back**								
Superior parietal lobe	7	10194	R	14	-68	58	8.92	<.001
Superior occipital gyrus	19		R	34	-78	30	7.41	
Middle temporal lobe	21		R	60	-48	-10	7.36	
Medial frontal gyrus/AC	6	1680	R	2	12	52	7.46	<.001
Superior-middle frontal gyrus	8		R	30	8	56	6.50	
	6		R	38	2	54	6.43	
Middle frontal gyrus (includes DLPFC)	8	1166	R	36	36	46	7.35	<.001
	9		R	44	40	36	5.80	
	10/46		R	36	52	14	5.52	
Middle frontal gyrus	8	862	L	-44	-2	50	6.78	<.001
Inferior frontal gyrus	44		L	-46	8	26	5.48	
Middle frontal gyrus (includes DLPFC)	46	399	L	-46	38	28	6.15	.015
	9		L	-42	32	38	6.01	
**2-back > 0-back**								
Precuneus	7	12114	L	-10	-62	56	13.22	<.001
Inferior parietal lobe	7		R	30	-70	46	12.58	
Superior parietal lobe	7		R	14	-68	56	11.83	
Middle frontal gyrus (includes DLPFC)	8	3768	R	26	12	54	9.24	<.001
	8		R	34	32	48	9.03	
	9		R	42	6	46	8.78	
Middle frontal gyrus (DLPFC)	9	426	L	-42	32	38	6.77	.016
Middle frontal gyrus	8	823	L	-38	0	52	6.49	.001
AC	32/6		L	-26	6	50	5.05	
Inferior frontal gyrus	44		L	-44	6	26	4.56	
Insula	n/a	322	R	36	18	-4	6.27	.043
			R	26	26	-2	4.89	
**0-back > 1-back**								
Posterior cingulate	23/31	2596	L	-8	-50	22	9.94	<.001
	31/24		R	2	-16	44	6.72	
Medial prefrontal gyrus	9	3664	L	-6	60	14	9.22	<.001
	9		L	-10	56	34	6.53	
	9		R	10	56	34	6.21	
Middle-inferior temporal gyrus	21	412	R	44	-10	-10	5.52	.013
	21/20		R	40	-12	-26	3.80	
**0-back > 2-back**								
Medial prefrontal gyrus	9	3374	L	-10	58	30	10.36	<.001
	10		L	-8	58	16	8.50	
	10		L	-4	50	-4	7.28	
Posterior cingulate	29/30	4867	L	-6	-48	22	9.49	<.001
Paracentral lobule	5		R	2	-22	54	8.74	
Posterior cingulate	29/30		R	8	-50	18	7.29	
Post-central gyrus	43	1592	R	38	-16	18	6.82	<.001
Insula	n/a		R	36	-16	8	5.95	

Pre-central gyrus	6		R	64	-2	14	5.71	
Post-central gyrus	43	817	L	-40	-16	16	6.76	.001
Parahippocampal gyrus	36		L	-28	-26	-24	4.67	
Insula/Middle temporal gyrus	21		L	-32	-18	0	4.22	
**Rest > 0-back**								
Hippocampus/ Parahippocampal gyrus	n/a	1117	R	20	-32	-4	8.29	<.001
Posterior cingulate	29		R	8	-46	6	4.97	
	29/30		L	-6	-48	10	4.27	
Precuneus	7	847	R	8	-82	40	7.68	<.001
	7		R	14	-90	42	6.97	
	19		L	-10	-92	38	6.08	
Insula	n/a	442	R	38	-14	12	6.75	.011
Superior-middle temporal gyrus	22/21		R	38	-4	-8	3.94	
Lingual gyrus	18	510	L	-24	-88	-14	6.30	.006
Lingual gyrus/Cuneus	17		L	-20	-100	-6	5.62	
Fusiform gyrus	18		L	-32	-82	-24	5.23	
Lingual gyrus	18	318	R	2	-80	-4	5.77	.036
Fusiform gyrus	18/19	335	R	26	-82	-20	5.64	.030
Lingual/Inferior occipital gyrus	17/18		R	24	-92	-14	5.53	
Cuneus/Inferior occipital gyrus	17/18		R	24	-98	-6	5.04	
Precuneus	7	586	R	2	-38	46	5.64	.003
Posterior cingulate	31		R	14	-48	38	4.91	
Middle temporal gyrus	21	366	L	-46	-6	-16	5.02	.022
	21		L	-50	0	-30	4.52	
	21		L	-40	-14	-4	4.46	
**Rest > 1-back**								
Posterior cingulate	23/30	12903	L	-6	-58	20	10.82	<.001
Middle temporal gyrus	21		R	42	-10	-16	10.02	
Insula	n/a		R	40	-16	12	8.90	
Medial prefrontal gyrus	9	3811	L	-12	54	34	9.20	<.001
	9		L	-10	46	36	8.54	
	9/10		R	10	58	24	7.59	
Fusiform gyrus	18	535	L	-32	-82	-24	6.67	.006
Lingual gyrus	18		L	-24	-88	-14	5.97	
Middle occipital gyrus	18/19		L	-20	-98	10	5.61	
**Rest > 2-back**								
Posterior cingulate	31	9078	L	-8	-50	30	23.03	<.001
	23/31		R	8	-52	24	14.13	
	31		R	2	-20	40	9.85	
Insula	n/a	4030	R	38	-14	10	13.11	<.001
Medial temporal lobe/Amygdala	28		R	28	6	-26	5.46	
Parahippocampal gyrus	35/36		R	34	-14	-30	4.98	
Medial prefrontal gyrus	9	3162	L	-12	56	32	9.89	<.001
	10		L	-2	58	8	9.04	
	10		L	-2	54	16	8.93	
Angular gyrus	39	342	L	-56	-66	34	7.06	.035
Middle temporal gyrus	39		L	-54	-72	26	6.60	
Occipital gyrus	18	410	L	-22	-100	-4	6.70	.018

Middle occipital gyrus	18	885	L	-26	-100	4	5.57	<.001
Fusiform gyrus	18		L	-32	-82	-24	5.24	
Middle temporal gyrus	21		L	-40	-16	-6	6.51	
Transverse temporal gyrus	41		L	-40	-16	16	6.46	

BA, Brodmann area; MNI, Montreal Neurological Institute; AC, anterior cingulate; DLPFC, dorsolateral prefrontal cortex.

Supplemental Table 2. Brain regions with significantly co-varying increases or decreases in activity (height threshold *p* =.005) in the left dorsolateral prefrontal cortex during the 2-back' (<0-back) condition in patients (CBT+TAU) and healthy participants.

**Patients**	BA	Cluster size (voxels)	Side	MNI Coordinates			Voxel T value	Cluster *p* (corrected)
				X	Y	Z		
**Regions with Positively Correlated Activity**								
Middle frontal gyrus (extends to include AC and right DLPFC)	46	19066	L	-48	34	28	19.55	<.001
	46		L	-44	48	8	10.52	
	10/46		L	-48	44	16	8.85	
Inferior parietal cortex	40	1144	L	-54	-46	54	4.54	.027
	40		L	-58	-54	42	4.14	
	40		L	-34	-50	48	3.87	
**Regions with Negatively Correlated Activity**								
Posterior cingulate	29/30	574	R	2	-46	20	5.31	0.22
	23/30		R	6	-56	10	2.98	(uncorrected .02)
**Healthy Participants**								
**Regions with Positively Correlated Activity**								
Middle frontal gyrus (extends to include AC and right DLPFC)	46	20441	L	-48	34	32	21.75	<.001
	10		L	-36	52	26	14.63	
	46		R	42	35	34	13.76	
Inferior-superior parietal cortex	7	1144	R	32	-84	40	10.27	<.001
	7		L	-38	-70	50	8.76	
	7		L	-30	-86	28	8.31	
**Regions with Negatively Correlated Activity**								
Posterior cingulate	23/31	4071	R	4	-50	24	5.95	<.001
	23/30		L	-14	-56	10	5.72	
	23/31		L	-8	-62	8	4.43	

BA, Brodmann Area; MNI, Montreal Neurological Institute; AC, anterior cingulate; DLPFC, dorsolateral prefrontal cortex.

Supplemental Table 3. Brain regions with significantly co-varying increases or decreases in activity (height threshold *p* =.005) in the right dorsolateral prefrontal cortex during the 2-back' (<0-back) condition in patients (CBT+TAU) and healthy participants.

**Patients**	BA	Cluster size (voxels)	Side	MNI Coordinates			Voxel T value	Cluster *p* (corrected)
				X	Y	Z		
**Regions with Positively Correlated Activity**								
Middle-inferior frontal gyrus (extends to include AC and left DLPFC)	45/46	35481	R	54	24	22	12.41	<.001
	44		R	46	8	34	8.43	
	45/44		R	46	44	2	7.96	
**Regions with Negatively Correlated Activity**								
None even at uncorrected level								
**Healthy Participants**								
**Regions with Positively Correlated Activity**								
Middle frontal gyrus (extends to include AC and superior parietal cortex bilaterally)	45/46	40347	R	50	22	20	17.22	<.001
	44		L	-40	6	34	10.83	
	9		L	-42	14	34	10.52	
**Regions with Negatively Correlated Activity**								
Fusiform gyrus (extends to parahippocampal gyrus)	37	3676	R	14	-56	-16	5.31	<.001
Posterior cingulate	29/30		L	-4	-56	4	4.58	

BA, Brodmann area; MNI, Montreal Neurological Institute; AC, anterior cingulate; DLPFC, dorsolateral prefrontal cortex.
